# Fail-Safe Transcriptional Termination for Protein-Coding Genes in *S. cerevisiae*

**DOI:** 10.1016/j.molcel.2009.07.028

**Published:** 2009-10-09

**Authors:** Ana G. Rondón, Hannah E. Mischo, Junya Kawauchi, Nick J. Proudfoot

**Affiliations:** 1 Sir William Dunn School of Pathology, University of Oxford, South Parks Road, Oxford OX1 3RE, UK

**Keywords:** DNA, RNA, PROTEINS

## Abstract

Transcription termination of RNA polymerase II (Pol II) on protein-coding genes in *S. cerevisiae* relies on pA site recognition by 3′ end processing factors. Here we demonstrate the existence of two alternative termination mechanisms that rescue polymerases failing to disengage from the template at pA sites. One of these fail-safe mechanisms is mediated by the NRD complex, similar to termination of short noncoding genes. The other termination mechanism is mediated by Rnt1 cleavage of the nascent transcript. Both fail-safe termination mechanisms trigger degradation of readthrough transcripts by the exosome. However, Rnt1-mediated termination can also enhance the usage of weak pA signals and thereby generate functional mRNA. We propose that these alternative Pol II termination pathways serve the dual function of avoiding transcription interference and promoting rapid removal of aberrant transcripts.

## Introduction

RNA polymerase II (Pol II) in *S. cerevisiae* transcribes genes for either polyadenylated mRNA or noncoding RNA such as small nuclear RNA (snRNA) and small nucleolar RNA (snoRNA). In both cases, Pol II termination is tightly coupled to RNA 3′ end formation, although different *cis*- and *trans*-acting elements are involved. Transcription termination of mRNA-encoding genes relies on recognition of the polyadenylation (pA) signal by cleavage/polyadenylation complexes that trigger Pol II disassembly from the template by a still-uncertain mechanism (Birse et al., 1998). According to the torpedo model, cleavage at the pA site provides an entry site for 5′ to 3′ exonucleases such as Rat1 that degrades Pol II-associated nascent RNA, destabilizing Pol II interaction with the template. Alternatively, the allosteric model proposes that transcription over the pA signal alters the processivity of elongating polymerases, making them prone to release from the template (Buratowski, 2005).

Termination of snoRNA and snRNA gene transcription in *S. cerevisiae* occurs by a different, specific mechanism involving the NRD complex (NRD) comprising Nrd1, Nab3, and Sen1 (Rasmussen and Culbertson, 1998; Steinmetz et al., 2001). Although the NRD termination mechanism is not fully understood, specific features of its three components have been characterized. Nrd1 binds to both a minimal RNA sequence GUA[A/G] and the Pol II Rpb1 carboxy-terminal domain (CTD) (Carroll et al., 2004; Conrad et al., 2000). The Nrd1 CTD-interacting domain (CID) is homologous to that of Pcf11, a component of CFIA. However, while Pcf11 binds preferentially to Ser2-phosphorylated (S2-P) CTD, Nrd1 interacts with Ser5-phosphorylated (S5-P) CTD (Vasiljeva et al., 2008). This difference in CTD specificity supports the role of Nrd1 in transcription termination on short genes and Pcf11 on longer mRNA-encoding genes (Gudipati et al., 2008; Vasiljeva et al., 2008). Nab3 binds to Nrd1 and RNA, showing specificity for UCUU sequence (Carroll et al., 2004; Conrad et al., 2000). Finally Sen1 is a putative RNA helicase from the SF1 super family and bears a DEAxQ consensus. Its ortholog in *S. pombe* has been shown to unwind nucleic acid hybrids in vitro (Kim et al., 1999). Sen1 also interacts with Pol II through Rpb1 CTD but lacks a CID (Ursic et al., 2004). Supporting its ubiquitous importance, NRD has also been shown to act on Pol II-derived cryptic unstable transcripts (CUTs; Wyers et al., 2005). Transcription termination of CUTs is coupled to RNA degradation by the nuclear exosome, a complex of exonucleases, assisted by TRAMP, which contains the poly(A) polymerase Trf4. As NRD interacts with TRAMP and the exosome, this is thought to recruit them to the 3′ end generated by NRD termination (Arigo et al., 2006; Thiebaut et al., 2006; Vasiljeva and Buratowski, 2006).

Rnt1 is an RNase III type endonuclease that recognizes a hairpin AGNN tetraloop, cleaving dsRNA at 14–16 nt from the loop (Chanfreau et al., 2000). This is followed by Trf4-dependent polyadenylation and exosome-mediated degradation (Allmang et al., 1999; Egecioglu et al., 2006). Simultaneously, Rat1 and Xrn1 target and degrade the 3′ product (Danin-Kreiselman et al., 2003). Rnt1-mediated cleavage is required for rRNA, snRNA, and snoRNA processing as well as degradation of unspliced or lariat RNAs (Allmang et al., 1999; Chanfreau et al., 1998; Danin-Kreiselman et al., 2003; Elela et al., 1996). It is also implicated in regulation of steady-state levels of mRNAs encoding iron metabolism proteins (Lee et al., 2005). Rnt1 cleavage is thought to occur cotranscriptionally, at least for Pol I-dependent transcription, as mutations in *RNT1* impair Pol I termination (Kawauchi et al., 2008; Prescott et al., 2004). In contrast, termination at snRNA and snoRNA genes is unaffected by deletion of *RNT1* (Kim et al., 2006).

We present evidence that malfunction of pA-dependent termination due to either mutations in *RAT1* or a weak pA signal triggers alternative termination pathways that re-establish Pol II termination. One of these fail-safe mechanisms corresponds to the previously characterized NRD termination pathway. The 3′ ends of readthrough transcripts generated in *rat1-1* strain match clusters of Nrd1- and Nab3-binding sites. The other fail-safe termination pathway is mediated by Rnt1 RNA cleavage. This provides an entry site for Rat1 and so promotes transcription termination. The upstream RNA is rapidly degraded by the exosome. However, if a weak pA site is close to the Rnt1 cleavage site (RCS), this is activated and the transcript is stabilized by Pap1-mediated polyadenylation. Finally we analyze the crosstalk between pA-dependent, NRD- and Rnt1-induced termination pathways in the endogenous *NPL3* locus and on reporter plasmids.

## Results

### NRD Induces Pol II Termination in *rat1-1* Mutant

**Figure 1:**
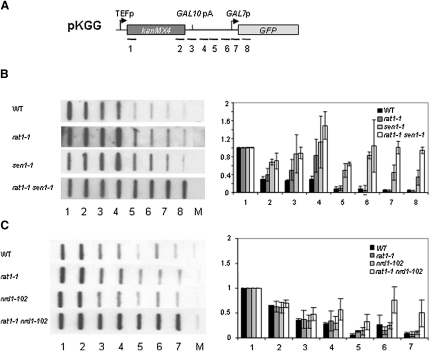
NRD Terminates Pol II on mRNA-Coding Genes in *rat1-1* Mutant (A) Diagram of pKGG employed in TRO. M13 probes used are indicated. Arrows denote *TEF1* and *GAL7* promoters and vertical line *GAL10* pA signal. (B) TRO and quantitation of WT, *rat1-1*, *sen1-1*, and *rat1-1 sen1-1*. M is background signal. Values are plotted relative to probe 1. Quantitations are based on three independent experimental repeats. Error bars show standard deviation. (C) WT, *rat1-1*, *nrd1-102*, and *rat1-1 nrd1-102* TRO in pKGG are as in (B).

We have shown that Sen1 cooperates with Rat1 to promote Pol II transcription termination on two different mRNA-coding genes, *CYC1* transcribed from the *GAL1* promoter, and the endogenous *PMA1* gene (Kawauchi et al., 2008). To extend this analysis, we performed transcription run ons (TROs) using the transcription termination reporter plasmid pKGG (*KanMX4*, *GAL10-*7 intergenic, *GFP*; Figure 1A; Morillon et al., 2003). Transcription from the pKGG *TEF* promoter was detected with strand-specific M13 probes spanning the construct (Figure 1A). As in later TROs, we show quantitation of triplicate experiments adjacent to a representative filter. Although TROs are subject to some variability, the profiles obtained are reproducible. In WT cells, *KanMX4* transcription terminated heterogeneously beyond probe 4. Slight variation in growth conditions altered the levels of 5′ end signal (probe 1), possibly reflecting promoter-restricted Pol II. While the single *rat1-1* mutant showed little increase in transcription beyond the poly(A) signal, the single *sen1-1* mutant did show some increase over probes 4–8, indicating a defect in Pol II termination. Higher readthrough levels were detected on the double *rat1-1 sen1-1* mutant (Figure 1B), indicating a more severe termination defect consistent with previous results (Kawauchi et al., 2008). To test if the *sen1-1* TRO phenotype was representative of other NRD components, we combined the *rat1-1* allele with *nrd1-102*, which is known to be defective in snoRNA gene termination (Conrad et al., 2000; Steinmetz et al., 2001). TRO analysis shows that while *nrd1-102* did not appreciably affect termination, *rat1-1 nrd1-102* double mutant generated a strong defect with Pol II transcribing into the downstream *GFP* gene (Figure 1C), similar to *rat1-1 sen1-1* (Figure 1B). The *rat1-1 nrd1-102* termination defect was confirmed using a different gene construct, pGCYC1 (data not shown). Together, these results indicate that, in the absence of functional Rat1, NRD acts to terminate readthrough transcription.

### Nrd1 Accumulates at the 3′ Ends of Protein-Coding Genes in *rat1-1* Mutant

**Figure 2:**
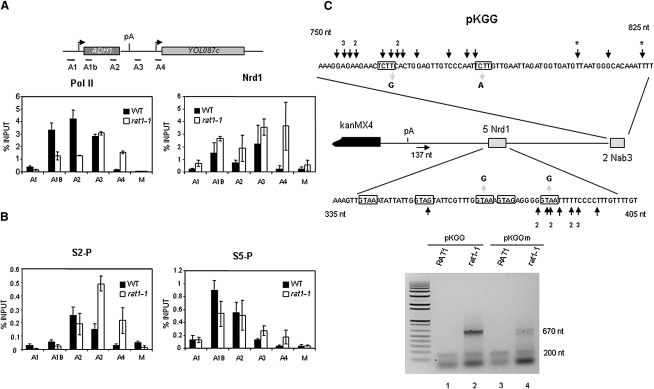
Nrd1 Accumulates in Intergenic Regions in *rat1-1* Mutants (A) Diagram of *ADH1*-*YOL087c*. Horizontal bars denote the ChIP probes. ChIP analyses using either anti-Rpb1 (8WG16) or IgG fast flow agarose were performed in TAP-*NRD1 RAT1* (WT) or TAP-*NRD1 rat1-1*. M corresponds to nontranscribed *MUC11*. Values are based on three independent ChIPs, and error bars show standard deviation. (B) ChIP with anti-S2-P CTD (3E10) or anti-S5-P CTD (3E8) on FD4D (WT) or FD4B (*rat1-1*) chromatin. (C) *GAL10-7* intergenic region in pKGG. pA signal is shown as a vertical line and Nrd1 and Nab3 clusters as gray boxes. Nrd1- and Nab3-binding sites (boxed) are shown as expanded sequences. Arrows indicate 3′ ends of readthrough transcripts determined by LM-RT-PCR. Multiple transcripts terminated at the same point are indicated by number, and transcripts adenylated are indicated by asterisk. Internal primer used in PCR (horizontal arrow) and position relative to *KanMX4* stop codon are indicated. Numbering at borders of the cluster sequences relate to stop codon position. Point mutations in pKGGm at the Nrd1 or Nab3 sites are indicated by gray arrows. Lower agarose gel shows the LM-RT-PCR products in *RAT1* and *rat1-1* strains depleted for the exosome (Rrp41). Estimated product sizes are indicated on the right. Lowest band corresponds to primer dimers.

Nrd1 has been shown to spread across the body of mRNA encoding genes, with a higher signal in the 5′ region suggesting a role in early elongation (Nedea et al., 2003). These data may conflict with its role in termination predicted by our above TRO results. We therefore investigated the possibility that Nrd1 profiles change in *rat1-1*. Chromatin immunoprecipitation (ChIP) analysis across endogenous *ADH1* in wild-type (WT) cells reveals Nrd1 signal spread across the whole gene peaking at the 5′ region and downstream of the pA signal (Figure 2A), as previously described (Nedea et al., 2003). In contrast with *rat1-1*, Nrd1 followed the WT distribution in the coding region but also accumulated in the 3′ flanking region (mainly over probe A4; Figure 2A, right panel). This profile mirrors Pol II distribution for *rat1-1* (Figure 2A, left panel) where higher readthrough signal in A4 indicates termination defects. Similar results were obtained on endogenous *PMA1* (see Figure S1 available online). From these experiments we infer that Nrd1 is associated with polymerases that fail to terminate in *rat1-1* mutant cells.

Recent work has indicated that Nrd1 specifically binds to Pol II S5-P CTD. Moreover, this modification is necessary to elicit termination via Nrd1 (Gudipati et al., 2008; Vasiljeva et al., 2008). We therefore tested the CTD phosphorylation state on polymerases that fail to terminate in *rat1-1*. ChIP analysis with S2-P (3E10) or S5-P (3E8) CTD-specific antibodies was performed on *ADH1*. As shown above, shifting *rat1-1* cells to nonpermissive temperature resulted in Pol II readthrough signals over probes A3 and A4 (Figure 2A). These polymerases were highly phosphorylated in Ser2 (Figure 2B, left panel). In addition, the rate of Ser5 dephosphorylation in *rat1-1* is lower than in the WT, resulting in similar levels of S5-P at the 5′ and 3′ ends of *ADH1* (A1B and A2) and higher levels than in the WT at A3 and A4 (Figure 2B, right panel). These results were confirmed in the *PMA1* locus (Figure S1). Overall we show that readthrough Pol II contains significant levels of S5-P CTD colocalized with Nrd1. These data support an additional function for NRD in terminating Pol II transcription when Rat1-dependent termination is impaired.

### Pol II Utilizes Nrd1- and Nab3-Binding Sites Downstream of Gal10 pA in a *rat1-1* Mutant

Nrd1 and Nab3 bind cooperatively to clusters of the minimal sequences GUA[A/G] and UCUU, respectively, which act redundantly to induce Pol II termination on snoRNA and snRNA genes (Carroll et al., 2007). pKGG employed in our TRO assays (Figure 1) contains two discrete clusters of these sequences downstream from the *GAL10* pA signal: five Nrd1-binding sites about 370 bp and two Nab3-binding sites about 760 bp downstream of the stop codon (Figure 2C). To determine if NRD-mediated termination observed in *rat1-1* correlates with Nrd1- and Nab3-binding sites, we performed ligation-mediated RT/PCR to map the 3′ ends of readthrough transcripts. Since these transcripts are unstable, we also employed a *GAL1p::RRP41* allele to deplete the exosome (Torchet et al., 2002). LM-RT-PCR analysis in *rat1-1* showed two discrete ends coinciding with the Nrd1- and Nab3-binding clusters (Figure 2C, lane 2). The lower band was also present in WT cells (Figure 2C, lane 1) and could therefore reflect RNA 3′ ends generated by pA-directed termination, as well as by NRD termination signals. We then cloned and sequenced *rat1-1* LM-RT-PCR products (39 individual clones). Thirteen clones had 3′ ends in the first cluster region around the fifth Nrd1-binding site, while 19 were in the second cluster mainly at the first UCUU Nab3 sequence. A further five clones mapped around 70 nt downstream of the last UCUU repeat. The precise sites are indicated by arrows (Figure 2C) except for those mapping to downstream Nab3 sites. Only two clones mapped between the two clusters. Five of the clones contain two to four extra adenines not present in the genomic sequence. Two are indicated with an asterisk in Figure 2C, and the other three are outside the clusters, one in the intermediate region and two downstream the second cluster. We predict that these nontemplated adenines are remnants of a short poly(A) tail added by TRAMP to promote exosome degradation (LaCava et al., 2005) and may correspond to the original 3′ ends generated by NRD termination. The other 3′ ends mapping near to the Nrd1- or Nab3-binding sites may be generated by partial degradation in the presence of low levels of exosome still remaining after metabolic depletion.

We mutated two Nrd1- and the two Nab3-binding sites as indicated (pKGGm) to evaluate their impact on termination. Mutations in the second cluster clearly reduced the number of transcripts ending in this region, as shown by a decrease in the intensity of the 670 bp PCR product (Figure 2C, lanes 2 and 4). Mutations introduced in the first cluster do not significantly alter its efficiency possibly due to the three residual Nrd1-binding sites and pA-dependent termination also occurring in this region. We predict from our results that these redundant Nrd1- and Nab3-binding sites cooperate to induce termination in this *GAL10* 3′ flanking region in *rat1-1* cells.

### Rnt1 Cleavage Sequence Terminates Pol II Transcription in the Absence of a pA Signal

**Figure 3:**
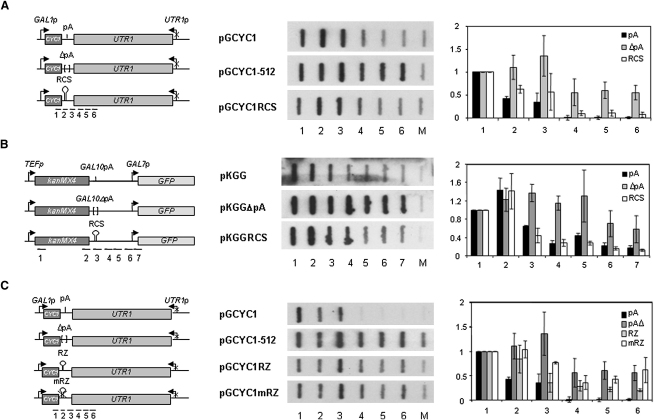
RCS Induces Pol II Termination When pA Is Deleted (A) Diagram of pGCYC1, pGCYC1-512, and pGCYC1RCS with *CYC1* promoter replaced by inducible *GAL1* promoter. The vertical line represents pA signal, brackets pA deletion and hairpin RCS. TRO were performed in W303 transformed with each plasmid. Hybridization profiles and quantitations are shown as in Figure 1. (B) Diagram of pKGG, pKGGΔpA, and pKGGRCS. *TEF1* and *GAL7* promoters are indicated by arrows, and pA, ΔpA, and RCS are as in (A). TRO was using W303 and shown as in Figure 1. (C) Diagram of pGCYC1, pGCYC1-512, pGCYC1RZ, and pGCYC1mRZ is as in (A), but the hairpin indicates RZ and crossed hairpin mRZ. TRO was performed in W303 and shown as in Figure 1. TRO quantitation error bars show standard deviation.

Although NRD may provide a fail-safe termination mechanism, the main termination pathway for protein-coding genes relies on the pA signal. The role of this signal in termination remains controversial, as there is evidence in support of both the torpedo and the allosteric models (Buratowski, 2005). To further discriminate between these two models, we generated a system whereby the *CYC1* pA signal is replaced by a strong RCS of 81 nt derived from rDNA repeats downstream the 25S gene. Initially RCS was cloned into the pGCYC1-512 plasmid in place of the deleted pA signal (pGCYC1RCS; Figure 3A). While Pol II transcribes beyond the site of normal transcription termination in pGCYC1−512, shown by signal over probes 4–6, RCS partially rescued this defect, decreasing the amount of signal over these regions (Figure 3A). To confirm this result, we cloned the RCS into pKGG with a deleted *GAL10* pA signal (pKGGΔpA) to form a new plasmid, pKGGRCS (Figure 3B). In agreement with previous studies on *GAL10-GAL7* (Greger and Proudfoot, 1998), deletion of the 55 nt encompassing the pA signal induces a substantial defect in termination shown by TRO as an increase in signal over probes 4–7. Again, insertion of RCS into pKGGΔpA restores termination, as shown by a reduction in probes' 3–7 signal (Figure 3B). We next considered the possibility that any cleavage in the RNA might suppress termination defects resulting from deletion of a pA signal. To test this hypothesis, we cloned a hammerhead ribozyme (RZ) or noncleaving mutant (mRZ) at the same position as the RCS in pGCYC-512 to generate pGCYC1RZ and pGCYC1mRZ, respectively. TRO analysis of these transformed plasmids shows that although the RZ slightly diminished the pGCYC1-512 termination defect, it does not suppress it (Figure 3C). We confirmed that RNA was cleaved in the presence of pA, RCS, and RZ but not mRZ by RT-PCR with primers flanking the cleavage positions (data not shown). These data imply that RCS, but not other cleavage events, leads to Pol II transcription termination in the absence of a canonical pA signal.

### Rnt1 and Rat1 Are Required for Termination Mediated by RCS

**Figure 4:**
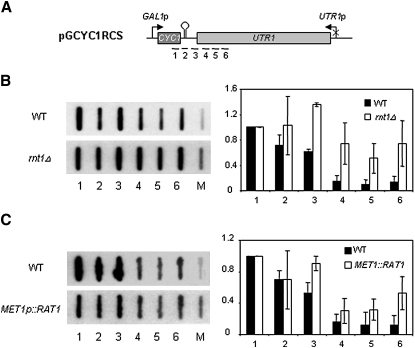
RCS-Dependent Termination Involves Rnt1 and Rat1 (A) Diagram of pGCYC1RCS employed for TRO as in Figure 3A. (B) BMA64 and *rnt1Δ* were used in TRO assay. Hybridization profiles and quantitation are presented. (C) As in (B), except W303 and YAEH97 were grown in media without methionine, and depletion was induced as described in the Experimental Procedures. TRO quantitation error bars show standard deviation.

To obtain mechanistic data on the process of RCS-induced termination, we investigated its dependence on Rnt1 activity. By TRO we observed that *RNT1* deletion significantly impairs termination in the pGCYC1RCS plasmid. In an isogenic WT strain, transcription largely terminated in region 3, in contrast to the *rnt1Δ* strain, where higher levels of Pol II transcription extended into region 6 (Figure 4B). This result argues for a role of Rnt1 in RCS-dependent termination but does not discriminate between a role in RNA cleavage or recruitment of other interacting factors, such as Sen1 (Ursic et al., 2004). However, when the *sen1-1* strain was transformed with pGCYC1RCS and termination measured at nonpermissive temperatures, the TRO profile was identical to WT (data not shown). This argues against a role for Rnt1 in recruiting Sen1 to mediate termination. To establish that RCS generates an entry site for Rat1, we performed TRO analysis on a strain bearing a methionine-repressible *RAT1* gene (*MET1p::RAT1*). This depletes Rat1 from the cell after 20 hr growth in media with methionine (El Hage et al., 2008). Under these conditions, a significant proportion of Pol II failed to terminate in region 3, resulting in increased signal over probes 4–6 as compared to isogenic WT (Figure 4C). The above results indicate that termination induced by RCS follows a similar mechanism to pA-mediated termination by supplying Rat1 with an entry site to degrade the RNA and consequently destabilize elongating Pol II.

### RCS-Mediated Termination Generates Unstable RNAs

**Figure 5:**
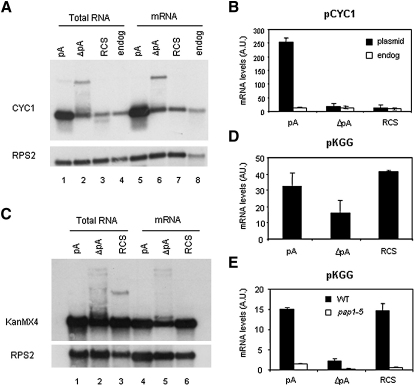
RNA Cleavage by Rnt1 Promotes Degradation Unless Close by Weak pA Site (A) Northern blot analysis of pGCYC1 (pA), pGCYC1-512 (ΔpA), pGCYC1RCS (RCS), and endogenous *CYC1* total and polyadenylated RNA from W303. *RPS2* RNA levels were used as loading control. (B) qRT-PCR from W303 transformed with pGCYC1, pGCYC1-512, pGCYC1RCS, and grown in galactose (plasmid) or glucose (endog) conditions. OligodT-primed cDNA was a template for real-time PCR using coding region *CYC1* primers. (C) Northern blot analysis of pKGG (pA), pKGGΔpA (ΔpA), pKGGRCS (RCS) total or pA selected RNA. Membranes were first hybridized with KanMX4 probe and subsequently stripped and reprobed for RPS2 RNA as control. (D) qRT-PCR from W303 transformed with pKGG, pKGGΔpA, and pKGGRCS. OligodT-primed cDNA from total RNA was used as a template for quantitative PCR with KanMX4-specific primers. (E) qRT-PCR from W303 and LM98 (*pap1-5*) transformed with pKGG, pKGGΔpA, and pKGGRCS and grown at 37°C for 2 hr to induce the mutation. Quantitation of mRNA was as in (D). Quantitation in (B), (D), and (E) shows error bars based on standard deviation.

The possibility that RCS-mediated Pol II termination provides a second fail-safe termination mechanism was further investigated. We therefore performed steady-state analysis to measure mRNA levels from RCS-terminated gene constructs (Figure 5). To control for the levels of endogenous *CYC1* mRNA expression, we isolated RNA from cells grown in glucose, since pGCYC1 is only expressed under galactose induction. Northern blot analysis of total or polyadenylated RNA shows high *CYC1* mRNA levels when expression from pGCYC1 is induced (Figure 5A). Deletion of the pA signal (ΔpA) or insertion of RCS largely abolished mRNA expression from pGCYC1 (leaving only residual endogenous *CYC1* mRNA), presumably because nascent RNA is rapidly degraded. The upper band observed in ΔpA corresponds to a cryptic pA site previously reported for pGCYC1-512 (Zaret and Sherman, 1984). This band was absent in lanes 3 and 7 (Figure 5A), confirming that RCS terminates transcription before Pol II reaches the cryptic pA. These results were repeated by quantitative (q) RT-PCR with oligodT on total RNA from cells transformed with pGCYC1, pGCYC1−512, or pGCYC1RCS (Figure 5B). Taken together, these observations suggest that Rnt1-dependent cleavage of the nascent RNA causes destabilization of the transcript, most probably by forming a 3′ end accessible to the exosome. Importantly, these experiments prove that there is no cryptic pA site in pGCYC1-512 that promotes termination in pGCYC1RCS. Hence RCS constitutes a termination pathway alternative to the pA signal and NRD-dependent pathways previously described (Kim et al., 2006). RCS-mediated termination shares components with pA signal-dependent termination, since both require Rat1 (Figure 4C; Kim et al., 2004). However, RCS cleavage fails to recruit poly(A) polymerases that might stabilize the RNA.

### RCS Enhances the Usage of Closely Positioned Cryptic pA Sites

In parallel with steady-state analysis of pGCYC1RCS, we assessed mRNA accumulation in the pKGG-based system used above for termination assays (Figure 1). Even though *GAL10* pA deletion precluded termination (Figure 3B), it only halved *KanMX4* mRNA levels (Figures 5C and 5D). This implies that deletion of the main *GAL10* pA signal results in selection of a weaker pA signal, still able to trigger RNA 3′ end formation but not inducing transcription termination. RCS insertion in pKGGΔpA rescued mRNA levels to pKGG values. We assayed whether transcript polyadenylation in the presence of RCS depends on the canonical pA polymerase Pap1. We therefore employed a *pap1-5* allele that is defective in polyadenylation at nonpermissive temperature to isolate RNA from the pKGG, pKGGΔpA, and pKGGRCS plasmids. qRT-PCR shows a strong reduction in *KanMX4* mRNA accumulation with all three constructs (Figure 5E). In contrast, deletion of *TRF4*, the TRAMP poly(A) polymerase, did not alter levels of *kanMX4* mRNA (data not shown). These results show that RCS can enhance utilization of cryptic pA sites.

### NRD Competes with Weak pA Sites to Elicit Pol II Termination

**Figure 6:**
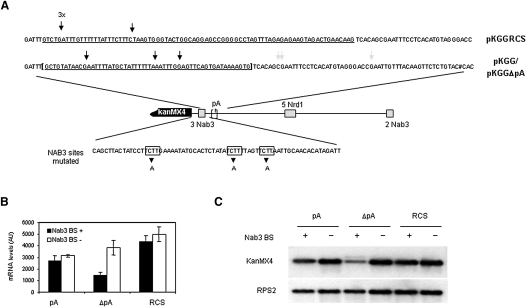
Nab3-Binding Sites Compete with a Weak pA Signal for 3′ End Formation (A) Diagram of *GAL10-GAL7* intergenic region in pKGG. Nrd1 and Nab3 sites are shown as gray boxes and pA site as a vertical line. pKGGRCS and pKGG sequences with the RCS and deleted pA signal (denoted by brackets) in pKGGΔpA are underlined. Arrows denote the 3′ ends mapped by 3′RACE in pKGG, pKGGΔpA (gray arrows), and pKGGRCS (upper sequence). Three Nab3-binding sites located upstream of pA are below the diagram. Arrowheads show the point mutations introduced in pKGGmNB3, pKGGΔpAmNB3, and pKGGRCSmNB3. (B) qRT-PCR from W303 was transformed with pKGG, pKGGΔpA, pKGGRCS (NB3), pKGGmNB3, pKGGΔpAmNB3, or pKGGRCSmNB3 (mNB3) as in Figure 5D. (C) Northern blot analysis of pKGG (pA), pKGGΔpA (ΔpA), pKGGRCS (RCS), with or without Nab3-binding sites upstream pA. Hybridization of pA-selected RNA was performed as in Figure 5C. Error bars show standard deviation.

To further characterize the cryptic pA sites remaining in pKGGΔpA, we performed 3′RACE on total RNA prepared from a WT strain transformed with this plasmid or pKGG. As previously described, *GAL10* mRNA displayed heterogeneous 3′ ends over the poly(A) signal region (Greger and Proudfoot, 1998). With the ΔpA sequence, three independent 3′ ends were placed within the region immediately downstream of the deleted pA site (gray arrows in Figure 6A). Strikingly, RCS insertion changed the polyadenylation site to the Rnt1 cleavage point or 20 nt upstream (Figure 6A, arrows in the upper sequence). We suggest that Rnt1 cleavage kinetics are faster than those of the residual poly(A) signal present in *GAL10* ΔpA. In some cases the 3′ end formed by Rnt1 cleavage is directly polyadenylated, but more often it is partially trimmed before being protected by a pA tail.

The analysis of the sequence upstream the weak pA signal present in pKGGΔpA reveals three Nab3-binding sites. We wanted to check if these sites influence the usage of the weak pA signal. They were therefore mutated in pKGG, pKGGΔpA, and pKGGRCS (Figure 6A). Northern and qRT-PCR showed no difference in mRNA accumulation in the presence or absence of Nab3-binding site (BS+ versus BS−) when the strong *GAL10* pA site or RCS is in the vicinity. Importantly in pKGGΔpA, mutation of Nab3-binding sites significantly increased the amount of mRNA reaching WT levels (Figures 6B and 6C). Together, these results argue for a competition between NRD and pA-dependent pathways when the pA signal is weak. In the presence of a strong pA signal, termination is coupled to polyadenylation, and stable mRNA is produced. However, when there is a weak pA signal, NRD-mediated termination may compete to induce termination and recruit the exosome. In contrast, insertion of RCS enhances weak pA usage and diminishes NRD-mediated termination.

### RCS Constitutes a Fail-Safe Transcription Termination Mechanism in NPL3

**Figure 7:**
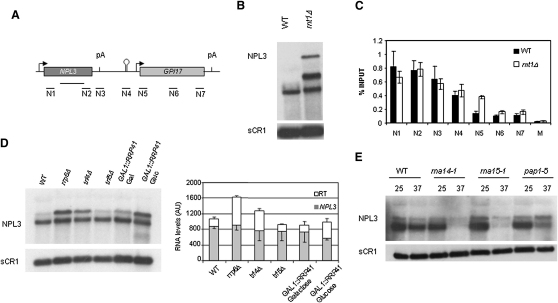
RCS Located Downstream of NPL3 Directs Termination and RNA Degradation (A) Chromosomal organization of *NPL3-GIP17*. Arrows denote promoters, hairpin the RCS, and vertical lines pA signals. ChIP probes (N1-7) and Npl3 northern probe are indicated. (B) Northern blot analysis of *NPL3* transcripts in BMA64 (WT) and *rnt1Δ*. (C) ChIP with anti-Rpb1 (8WG16) was performed in BMA64 (WT) and *rnt1Δ*. M denotes *MUC11* as negative control. Average of three independent experiments is shown, and error bars represent standard deviation. (D) Northern blot analysis of BY4741 (WT), Y01777 (*rrp6Δ*), Y06265 (*trf4Δ*), Y01145 (*trf5Δ*), and DRY41-4A (*GAL1::RRP41*) strains grown in glucose, except for DRY41-4A, which was grown in galactose or glucose for 20 hr to induce exosome depletion. Total RNA was assayed as in (B). *NPL3* transcripts from three independent experiments were quantified and normalized to the amount of sCR1. The average of the three values is represented in gray and the readthrough transcript (upper band in the gel) in white. Error bars show standard deviation. (E) Northern blot of W303 (WT), LM 88 (*rna14-1*), LM91 (*rna15-1*), and LM98 (*pap1-5*) grown at 25°C or shifted 2 hr at 37°C.

RCSs are broadly distributed in the yeast genome. Furthermore, Rnt1 substrates were found in precursors of rRNA, snRNA, and snoRNA and more recently in some coding and integenic regions (Allmang et al., 1999; Chanfreau et al., 1998; Danin-Kreiselman et al., 2003; Elela et al., 1996). We wished to check whether RCS present downstream of an endogenous mRNA-encoding gene can trigger transcription termination and also determine the fate of this transcript. The tandem *NPL3-GIP17* locus was therefore selected, as it contains an unusually long intergenic region that may fold into an AGNN tetraloop hairpin, as predicted by M-fold (Zuker, 1994). Furthermore, a similar analysis of *NPL3-GIP17* has identified a functional RCS between these two genes (Ghazal et al., 2009). By circular LM-RT-PCR, we mapped the *NPL3* pA site 250 nt downstream the stop codon (data not shown). However, 3 out of 16 clones analyzed were longer, indicating that a proportion of polymerases fail to terminate at the pA site. This is in agreement with recent studies showing that *NPL3* pA signal is weak and Npl3 itself regulates pA site usage by competing with 3′ end cleavage and polyadenylation factors (Lund et al., 2008). To determine whether the intergenic region is transcribed and cleaved by Rnt1, total WT and *rnt1Δ* RNA was analyzed by northern blot using a probe spanning the *NPL3* coding region. *RNT1* deletion yielded similar levels of *NPL3* mRNA to the WT. However, two additional major transcripts corresponding in size to readthough transcripts ending near the *GIP17* 5′ end and 3′ terminal pA site were also evident (Figure 7B). To investigate *NPL3-GIP17* transcription termination more closely, we performed Pol II ChIP analysis. Pol II signal was consistently high across the intergenic region of *NPL3-GIP17*, indicative of inefficient termination presumably due to a relatively weak *NPL3* pA signal. However, this signal reduced immediately following the RCS in region 5 to a level that correlates with the lower *GIP17* transcription profile (Figure 7C). In *rnt1Δ*, Pol II signal remained downstream of RCS, confirming a role for Rnt1 in transcription termination in region 5. Most of the polymerases that transcribe beyond the RCS terminated at the cryptic pA located at *GIP17* 5′ region (N6), although a small proportion still continued until *GIP17* pA site (Figures 7B and 7C). This suggests that a substantial proportion of Pol II transcribing *NPL3* fails to terminate at the pA site and continues into the RCS region. Rnt1 normally cleaves these readthrough transcripts, inducing termination and degradation of these aberrant RNAs. However, in the absence of Rnt1, Pol II terminates at a cryptic pA site located at the 5′ region of *GIP17* or at the *GIP17* natural pA site.

We next investigated the fate of *NPL3* readthrough RNA cleaved at the RCS. Previous studies on genes that contain RCS showed that Rnt1-cleaved RNA is rapidly degraded by Rat1 and the nuclear exosome (Allmang et al., 1999; Danin-Kreiselman et al., 2003). If this is also the case for *NPL3* readthrough transcripts, mutations in the exosome should display stabilizing effects. We visualized *NPL3* transcripts in conditions of metabolic depletion or deletion of different subunits of the exosome or the TRAMP complex (Figure 7D). The absence of functional exosome stabilized a longer Npl3 transcript ending at the RCS. This transcript was smaller than the readthrough transcripts observed in an *rnt1Δ* strain, consistent with the RCS location upstream *GIP17*. The amount of *NPL3* mRNA was not significantly affected in exosome mutants (Figure 7D), but it was strongly reduced in cleavage/pA mutants (Figure 7E), proving their requirement for pA and 3′ end processing factors in *NPL3* mRNA formation. Together these results reveal a dual role for the RCS at the *NPL3* locus. On one hand, it ensures Pol II termination, thereby minimizing transcription interference on *GIP17* that might otherwise occur due to the weak *NPL3* pA signal. In addition, it provides an entry site for the exosome that, aided by TRAMP, removes these aberrant transcripts.

Our analysis on *NPL3* confirms our above results on Pol II termination at the pGCYC1RCS and pKGGRCS plasmids. Overall, RCS constitutes an alternative, pA-independent termination pathway that is coupled to aberrant RNA degradation.

## Discussion

We describe the existence of two fail-safe transcription termination pathways for protein-coding genes. NRD previously associated with termination of snRNA, snoRNA, and CUT genes also induces termination of polymerases that read through a pA signal due to mutations in *RAT1*. Similarly, Rnt1-mediated Pol II termination is pA independent and requires Rnt1 and Rat1 activity. Since Rnt1-mediated RNA cleavage is not coupled to stabilization of the transcript by polyadenylation, the 5′ cleavage product is rapidly degraded by the exosome. However, if Rnt1 cleavage occurs in the vicinity of a weak pA site, then 3′ end processing factors are recruited and the transcript is polyadenylated by Pap1. A natural example of Rnt1-dependent termination is that of *NPL3*. Here Pol II termination is inefficient, and some polymerases transcribe beyond the pA site into the intergenic region containing a RCS. This induces transcription termination and degradation of the readthrough transcript.

Pol II transcription termination at protein-coding genes requires the coordination of multiple 3′ end formation factors. These may affect CTD S2-P levels, provide a permissive chromatin environment, promote recognition of pA signal by the 3′ end processing machinery, and subsequent Rat1-dependent Pol II termination. Such complexity may cause a proportion of polymerases to fail to terminate even in WT cells, resulting in transcriptional interference of tandem downstream genes (Greger and Proudfoot, 1998). Also with convergent genes, readthough polymerases may generate antisense transcripts that induce heterochromatin formation (Camblong et al., 2007; Gullerova and Proudfoot, 2008). We propose that the NRD- and Rnt1-mediated fail-safe mechanisms provide a second chance to terminate Pol II, and so minimize these deleterious effects.

### NRD Termination Pathway as a Fail-Safe Mechanism

In contrast to mammalian genes, yeast promoters of noncoding and protein-coding genes are similar, and no specific transcription factors have been described. Accordingly, Rat1 and NRD can both be found associated with Pol II transcribing either gene type (Kim et al., 2006; Nedea et al., 2003). Termination signals may be redundant, since an snRNA terminator cloned at the 3′ end of a protein-coding gene can elicit termination by generating a polyadenylated RNA (Steinmetz et al., 2006a). It seems likely that the molecular decision to terminate transcription is taken at the 3′ end of the gene and may be influenced by other factors than just the available *cis*-acting sequences. Here we show that combining mutations previously reported to affect termination of noncoding genes (*sen1-1* or *nrd1-102*) with a *rat1-1* allele causes a strong increase in mRNA encoding gene readthrough transcription. Significantly, these readthrough transcripts end at Nrd1- and Nab3-binding sites. Discrimination between pA- or NRD-dependent termination correlates with the phosphorylation state of Pol II CTD. Nrd1 binds preferentially to S5-P CTD, and S2-P antagonizes this interaction (Gudipati et al., 2008; Vasiljeva et al., 2008). In contrast, pA-mediated termination requires S2-P CTD (Ahn et al., 2004). These data are consistent with the fact that the distance between a promoter and a CUT influences termination efficiency (Gudipati et al., 2008; Kopcewicz et al., 2007) and that termination of the 1 kb polycistronic *SNR* is not affected by *SEN1* mutations (Steinmetz et al., 2006b). Notably, there is no clear limit for NRD-dependent termination but rather a gradual decrease in efficiency with a commensurate increase in pA-dependent termination (Gudipati et al., 2008). We now show that NRD-dependent termination can occur more than 1.5 kb downstream a promoter in the pKGG system when pA-dependent termination is impaired as shown in Figures 1 and 2. This effect may be favored by a slight decrease in Ser5 dephosphorylation observed in *rat1-1* mutants. Importantly, the level of S5-P needed to elicit NRD-dependent termination is low, as Pol II recognizes an NRD terminator at 600 bp downstream of the promoter where the amount of CTD S5-P is small (Ahn et al., 2004).

### Rnt1 Cleavage Triggers Pol II Termination

Rnt1 initially identified based on homology with bacterial RNase III principally acts to process precursor molecules of noncoding RNAs such as rRNA, snRNA, and snoRNAs. We previously showed that *RNT1* deletion causes Pol I to read through the rDNA termination region (Kawauchi et al., 2008; Prescott et al., 2004). Conversely, Pol II transcription termination at snRNA or snoRNA genes is not influenced by *RNT1* mutants (Kim et al., 2006). We now show that Rnt1-mediated transcript cleavage terminates Pol II when transcribing protein-coding genes. Thus a RCS placed downstream of a gene lacking pA signals suppresses otherwise defective termination. This constitutes an additional pA-independent termination mechanism to NRD. Significantly, Rnt1- or pA-dependent termination is equally robust as judged by TRO (Figure 3). Some Rnt1 sites are formed at internal positions in Pol II-transcribed noncoding genes (e.g., snRNA), where they act to facilitate RNA processing but do not promote Pol II termination (Allmang et al., 1999). Here NRD-mediated termination may be more efficient due to high S5-P CTD levels and the presence of strong Nrd1- and Nab3-binding sites. Also, the RCS derived from the Pol I terminator used in our experiments may bind Rnt1 with higher affinity than RCS present in snRNAs. Rnt1-induced termination involves Rat1, suggesting a torpedo-like mechanism. It is likely that other factors recruited specifically by Rnt1 or the RCS are involved, since cleaving the RNA with a ribozyme does not elicit termination. Sen1 interaction with Rnt1 (Ursic et al., 2004) is a possible candidate for facilitating Rnt1-mediated termination, either by ensuring that the RNA is properly folded into the tetraloop or by bringing NRD into play. However, our results indicate that Sen1 is not required for Rnt1-mediated termination. Further analysis is required to characterize the exact mechanism by which Rnt1 cleavage promotes Pol II termination.

### Rnt1-Mediated Termination Enhances Recognition of Weak pA Signals

The fate of a nascent transcript terminated by Rnt1 depends on neighboring signals. We observed that in the absence of a pA site, RNA is completely degraded upon Rnt1 cleavage due to Trf4 polyadenylation and exosome-mediated digestion (Allmang et al., 1999; Egecioglu et al., 2006). This constitutes a surveillance pathway to remove RNAs that fail to assemble (Morlando et al., 2004). Similarly, mRNAs that are not properly polyadenylated are rapidly removed by the exosome (Torchet et al., 2002). However, when a weak pA signal is close to the RCS, the RNA is fully polyadenylated and so escapes degradation. In the *GAL10* termination region, deletion of the major pA signal impairs termination and decreases polyadenylation of the transcript. However, this 55 nt deletion does not preclude 3′ end processing, as a significant amount of mRNA is still detected. Insertion of the RCS rescues termination and full levels of polyadenylation. This polyadenylation is mediated by Pap1, indicating that 3′ end factors are recruited. We predict that the remnant sequence in the *GAL10*ΔpA is still able to assemble 3′ end processing factors but that RNA cleavage is very inefficient. Insertion of RCS provides an alternative efficient mechanism to cleave the transcript.

*NPL3* encodes an RNA-binding protein involved in multiple stages of RNA metabolism and may act as a transcriptional antiterminator by competing with 3′ end processing factors for binding to RNA (Bucheli and Buratowski, 2005). Indeed, Npl3 autoregulates its own pA site usage. Thus, increased levels of Npl3 favor its binding to the pA site instead of CFIA. As a result, transcription proceeds into the intergenic region, generating unstable readthrough transcripts (Lund et al., 2008). Interestingly, Sen1 acts to enhance Npl3 autoregulation, since its inactivation causes a reduction in readthrough transcription (Steinmetz et al., 2006b). Possibly Sen1 helicase activity alters the structure of the *NPL3* transcript in favor of Npl3 recognition. Our results confirm that these readthrough transcripts cleaved by Rnt1 are substrates for TRAMP exosome-mediated degradation. This provides a natural example of Rnt1-mediated termination as a fail-safe mechanism. A proportion of polymerases read through the weak *NPL3* pA site into downstream signals where Rnt1 cleavage triggers termination. In addition, this Rnt1 cleavage process results in the appearance of an unprotected 3′ end favoring the rapid degradation of these aberrant RNAs. Mutation of 3′ end processing factors precludes pA usage, and as a consequence, more transcription is terminated via Rnt1, producing no stable mRNA. Interestingly, mutations in TRAMP or the exosome stabilize readthrough transcripts without increasing the amount of *NPL3* mRNA. This indicates that once the transcript is cleaved it can not be processed at the pA site. It is important to mention that Rnt1-mediated Pol II termination of protein-coding genes may be a relatively common process in *S. cerevisiae.* Thus, a parallel study (Ghazal et al., 2009, in this issue of *Molecular Cell*) using genome-wide analysis has revealed that several additional genes to *NPL3* generate 3′ flanking region Pol II profiles and detectible readthrough transcripts in *rnt1Δ* strains, indicative of Rnt1-dependent termination.

Overall, the data presented in these studies demonstrate the existence of three different termination pathways for protein-coding genes. The dominant pA-dependent mechanism exclusively generates stable mRNA. If Pol II fails to terminate at the pA site, then NRD- or Rnt1-mediated termination may act as a fail-safe mechanism. This remarkable triplication of Pol II termination mechanisms in *S. cerevisiae* must reflect strong evolutionary selection to maintain separate transcription units.

## Experimental Procedures

### Yeast Strains and Plasmids

Strains, plasmids, and oligonucleotides used in this study are listed in Tables S1–S3.

Yeast strains were grown in minimal media at 25°C and transferred to 37°C 2.5 hr prior to TRO or ChIP assays. YCBA63 and DRY41-4A were grown in selective media with 2% galactose to OD 0.6 and then transferred to selective media with 2% glucose for 20 hr at 25°C and 2.5 hr at 37°C. YAEH97 was grown at 30°C to OD 0.5 in SD medium lacking methionine, then diluted to OD 0.03 and grown for 20 hr in medium supplemented with methionine (5 mM).

### ChIP

ChIP was performed as described (Komarnitsky et al., 2000), with modifications presented in the Supplemental Data.

### TRO Analysis

TRO analysis and probes for pKGG, pGCYC1, and derived plasmids are as described (Birse et al., 1998; Morillon et al., 2003). Locations of the probes used are shown in Figures 1 and 3. Signals are plotted relative to probe 1 between WT and several mutant strains. Figures 1, 3, and 4 display data based on three repetitions.

### LM-RT-PCR, 3′RACE, and cRT-PCR

LM-RT-PCR was performed as described (Hitchcock et al., 2004) with modifications presented in the Supplemental Data.

3′RACE was performed with the primers listed in Table S3.

cRT-PCR was performed as previously described (Couttet et al., 1997), with 5 μg of total RNA from W303. The primers used for nested PCR are indicated in Table S3. PCR products were cloned and the resulting colonies screened as described above.

### Northern Blot and Q-RT-PCR

Northern blot was performed from 8 μg of total RNA or 400 ng of mRNA isolated with Oligotex mRNA kit.

qRT-PCR employed 1 μg of total RNA with oligodT and SuperScript III following the manufacture indications. Two microliters were used as template for each quantitative PCR, using primers as listed in Table S3.

## Supplemental Data

Document S1. Three Tables, Supplemental Experimental Procedures, Supplemental References, and One Figure
