# Supplementary material for: Fail-Safe Transcriptional Termination for Protein-Coding Genes in S. cerevisiae
Source: Mol Cell. 2009 Oct 9;36(1):88–98. doi: 10.1016/j.molcel.2009.07.028 (PMC2779338; doi:10.1016/j.molcel.2009.07.028)
Supplement: Document S1. Three Tables, Supplemental Experimental Procedures, Supplemental References, and One Figure [file mmc1.pdf]

Molecular Cell, Volume *36*

Supplemental Data

Fail-Safe Transcriptional Termination for Protein-Coding Genes in *S. cerevisiae*

Ana G. Rondón, Hannah E. Mischo, Junya Kawauchi, and Nick J. Proudfoot

Table S1. Yeast strains

**Strain**	**Genotype**	**Reference**
FD-4A	Matα *leu2 trp1Δ63 ura3*	(Kawauchi et al., 2008)
FD-4B	Mat *a leu2 ura3 rat1-1*	(Kawauchi et al., 2008)
FD-4C	Mat α *leu2 trp1Δ63 ura3 sen1-1*	(Kawauchi et al., 2008)
FD-4D	Mat *a leu2 ura3 rat1-1 sen1-1*	(Kawauchi et al., 2008)
JDY-1A	Mat *a his3 leu2 met15Δ0 ura3 rat1-1*	This study
JDY-1B	Mat α *leu2 trp1Δ63 ura3 rat1-1 nrd1-102*	This study
JDY-1C	Mat *a his3 leu2 met15Δ0 trp1Δ63 ura3 nrd1-102*	This study
JDY-1D	Mat α *leu2 ura3*	This study
YCBA63	Mat a *ade2 his3 leu2 trp1 ura3 HIS3-GAL::protA-RRP41*	(Torchet et al., 2002)
DRY41-4A	Mat α *leu2 trp1 ura3 rat1-1 HIS3-GAL::protA-RRP41*	This study
SC2290	Mat a *ade2 arg4 leu2-3,112 trp1-289 ura3-52 NRD1-TAP::URA3*	Euroscarf
SDY90B	MAT α *leu2 trp1 ura3-52 rat1-1 NRD1-TAP::URA3*	This study
W303-1A	Mat a *leu2-3 112 trp1-1 can1-100 ura3-1 ade 2-1 his3-1,*	R. Rothstein
LM88	W303-1A but *rnal4-1*	(Minvielle-Sebastia et al., 1994)
LM91	W303-1A but *rnal5-1*	(Minvielle-Sebastia et al., 1994)
LM98	W303-1A but *paplΔ::LEU2* pApapl-5.	(Minvielle-Sebastia et al., 1994)

YAEH97	W303-1A but P_{MET3}-*RAT1* (*HIS3*)	(El Hage et al., 2008)
BY4741	MAT a *his3Δ1 leu2Δ0 met15Δ0 ura3Δ0*	Euroscarf
Y01777	BY4741 but *RRP6::kanMX4*	Euroscarf
Y06265	BY4741 but *TRF4::kanMX4*	Euroscarf
Y01145	BY4741 but *TRF5::kanMX4*	Euroscarf
BMA64	MAT a *ura3-1 trp1 ade2-1 leu2-3,112 his3-11,15*	A. Jaquier
rnt1Δ	BMA64 but *rnt1::TRP1*	A. Jaquier

Table S2. Plasmids

pGCYC1	*CYC1* gene under GAL1 promoter	(Birse et al., 1998)
pGCYC1-512	pGCYC1 with 38 nt at *CYC1* pA site deleted	(Birse et al., 1998)
pGCYC1RCS	pGCYC1-512 with RCS cloned into *EcoRV* located 24 nt downstream *CYC1* stop codon	This study
pGCYC1RZ	pGCYC1-512 with RZ cloned into the same position as RCS in pGCYC1RCS (see Dye et al., 2006 for RZ and mRZ sequences)	This study
pGCYC1mRZ	pGCYC1-512 with mRZ cloned into the same position as RCS in pGCYC1RCS	This study
pKGG	KanMX4 gene under Tet promoter fused to *GAL10-GAL7* intergenic region and GFP	(Morillon et al., 2003)
pKGGΔpA	pKGG without 55nt encoding *GAL10* pA site (79 -134 nt downstream the stop codon)	This study
pKGGRCS	pKGGΔpA with RCS inserted 79 nt downstream KanMX4 stop codon	This study
pKGGm	pKGG with 2 Nrd1 and 2 Nab3 sites located downstream *GAL10* pA site mutated as indicated in figure 2C	This study
pKGGmNB3	pKGG with 3 Nab3 sites located upstream *GAL10* pA site mutated as indicated in figure 6A	This study
pKGGΔpAmNB3	pKGGΔpA with 3 Nab3 sites located upstream *GAL10* pA site mutated as indicated in figure 6A	This study
pKGGRCSmNB3	pKGGRCS with 3 Nab3 sites located upstream *GAL10* pA site mutated as indicated in figure 6A	This study

Table S3. Oligonucleotides

Primers used in qPCR (ChIP)

P0F	CCGGTGACGAAACGTGGTCG
P0R	CAACGAGGTTGATAGAAAAAAAAAGTTTTTGTAG
P2F	GACGACGAAGACAGTGATAACGATGGTC
P2R	CTTCTTTCTTCTTTTCAAAACTTCATCGGAAG
P5F	GGCTCCTGGTCTATCTGCTATTATTGATGC
P5R	CAATCCATAGACCCAAGAAGATTTCCAAATG
P6F	GAGCCAACAAGAATAAGCCGCTTATTTCC
P6R	GAAAAAGTACCATCCAGAGAAACCAATTATATCAAATC
P7F	GAATCGTCTTTATTATGGTCAAGGCTTTACGTC
P7R	GAGTGTCTGTATGGGCGCATAAACGTAAG
P8F	CGTAGTATTCGATGATTGAAGGCCGA
P8R	GAAAAGGAAACTTGTAGAATGGCCTGGC
P9F	GGCGAAATTTGCCCGGTTTTGTC
P9R	GAAAATTCAGCGGAAACAGCGTGATGAG
P10F	GGTTTACACTCCATCCAAGGGTCC
P10R	CTTCGAAAGCTTGTGGAGAGGTGAC
MF	CTAAGGAAAAGCCTACACCCCCAACC
MR	GGAGTAGTAGTCTTCTTAGTGCATGTCTTAGATGTGG
VF	GGCTGTCAGAATATGGGGCCGTAGTA
VR	CACCCCGAAGCTGCTTTCACAATAC
SNR13F	GGAAGTTTTTTCCTTTTTATATGATGAATATG
SNR13R	GGTCAGATAAAAGTAAAAAAAGGTAGCTTGAG
A1F	CGACAAAGACAGCACCAACAGATG
A1R	GAAGGAAGGCCGTATACCGTTGC
A1BF	GTGTTATCTTCTACGAATCCCACGGTAAG
A1BR	CCAAGCGTGCAAGTCAGTGTG
A2F	TTCAACCAAGTCGTCAAGTCCATCTCTA
A2R	ATTTGACCCTTTTCCATCTTTTCGTAA
A3F	CGGCATGCCGAGCAAATG
A3R	CCCAACTGAAGGCTAGGCTGTGG
A4F	CAAGTAACTACCAGCACCATAACACCG
A4R	GCAATCCAACTACACTTATACGACGTC
N1F	CAGTCGAAGAACAGCACCAAGAACCAC
N1R	GGACACTGTGGGATTCTTCGCCTTC
N2F	CCTAGAGGTGGTTACGATAGTCCAAGAG
N2R	GTTCTGTATGCATCTCTTGGAGGACC
N3F	GTGTTAGAAGATCCCAAAGTATATGATTGAAAACAG
N3R	GCACTCATCAATTTGGAAATGAAATTC
N4F	CAAGCCGATCTATTTTGGATATCTTATTGTC
N4R	CAATTGCACCTCTTAACTGGTACTTTATTTCTTG
N5F	GATAAGGATTACACGCCAGAATAATGTCC
N5R	GTGACTCAATGTAATTTATTGGTAGTGATGCTC
N6F	GTACAAGCGTTACGTGGTTTGATCTCTC
N6R	CGTCTGAAATCCGAGTGCCATTAATGAACG
N7F	CAGTCATGTTCTTTGGTTTCTACAACGTG
N7R	CTCATAATTCATCTTCACCATCAATAGCATG

Primers used in LM-RT-PCR (5' to 3')

RNA:DNA adapter	UUUCTCCATGTAACCTAGCC
Rev adapter	GGCTAGGTTACATGGAG
KGG-pA	CAACAAGAATATAGCACGAG

Primers used for site directed mutagenesis (5' to 3')

NR1mF	GTATTCGTTTGGGAAAGTAGAGGGGGGAATTTTTCCC
NR1mR	GGGAAAAATTCCCCCCTCTACTTTCCCAAACGAATAC
NB3mF	GAGAAGAACTCTGCACTGGAGTTGTCCCAATTATTGTTGAATTAG
NB3mR	CTAATTCAACAATAATTGGGACAACTCCAGTGCAGAGTTCTTCTC
NB3pAmF	CTATCCTTATTGAAAATATGCACTCTATATATTTTAGTTATTAATTGC
NB3pAmR	GCAATTAATAACTAAAATATATAGAGTGCATATTTTCAATAAGGATAG

Primers that encode RCS (5' to 3')

RCSF	GTCTGATTTGTTTTTATTTCTTTCTAAGTGGGTACTGGCAGGAGCCGGGGCCTAGTTTAGAGAGAAGTAGACTGAACAAG
RCSR	CTTGTTCAGTCTACTTCTCTCTAAACTAGGCCCCGGCTCCTGCCAGTACCCACTTAGAAAGAAATAAAAACAAATCAGAC

Primers used for 3´RACE and cRT-PCR

3RACE oligodt-adaptor	TTCGTATACCCGGGTACCAA$(T)_{17}$
3RACE Reverse	TTCGTATACCCGGGTACCAA
KanMXRACEF	CATCCTATGGAACTGCCTCGG
NPL3cRNAF1	CAGATTCTGGTAGTTGCTC
NPL3cRNAF2	CAAGAGGTAGCTACGGTGGTTC
NPL3cRNAR1	GATTATGGTCCTCCAAGAGA

Primers used for quantitative RT-PCR

CC1F	TGGCAGACACTCTGGTCAAG
CC1R	AAGGGGCCTGTTTACTCACA
KanMXF	CATCCTATGGAACTGCCTCGG
KanMXR	CAAGAAGGATAGTAAGCTGGCAAAGTC

Supplemental Experimental Procedures

Chromatin immunoprecipitation modifications

Sonication was performed in a Diagenode Bioruptor for 15 min at medium power. For each sample, equal amounts of protein were immunoprecipitated for 8-12 hrs at 4°C with either Fastflow IgG Sepharose or 8WG16, 3E10 or 3E8 antibodies coupled to Protein A agarose. Washes for Fastflow IgG Sepharose and 8WG16 were as described for H14 and for 3E10 or 3E8 as H5 washes (Ahn et al., 2004). Samples were treated with RNase A (30 min 37° C), and proteinase K while de-crosslinking (5 hrs at 65 °C) DNA was purified with Qiagen PCR-clean up columns and quantitated by real time PCR, using Sensimix with a Corbett Rotorgene 6000 lightcycler. IP signal was subtracted for signal derived from a no-antibody control and normalized to a serial dilution of the total chromatin. Sequences of the PCR primers employed are shown in Supplemental Table 3.

LM-RT-PCR

First, 0.25 nmol of the RNA:DNA adapter primer was 5'-kinased with T4 PNK and ATP. Total cell RNA (4 μg) was added to the 125 pmol kinased RNA adapter and treated with 20 U of T4 RNA ligase in a final volume of 20 μl at 16 °C for 1 h. Ligated RNA was phenol:chloroform purified and ethanol precipitated. The pellet was resuspended in 8 μl of reverse transcriptase (RT) reaction mix containing 3 μg of the reverse adapter primer, denatured at 85 °C 5 min and cooled to 50 °C, 1 μl RNase OUT and 1 μl Superscript III were added. The RT reaction was incubated for 30 min at 50 °C, followed by 20 min at 65°C. 2 μl cDNA was used as template in a 20 μl PCR amplified in 36 cycles. PCR products were treated with 1 μl of DNase free RNase I (Roche) and resolved on 1% agarose gels. DNA was cloned into the pCRII vector (TOPO TA Cloning Kit). Inserts were sequenced by Geneservice (Oxford UK). The location of the gene specific PCR primer is shown in Figure 2C and its sequence is in Supplementary Table 3.

Supplemental References

Ahn, S.H., Kim, M. and Buratowski, S. (2004) Phosphorylation of serine 2 within the RNA polymerase II C-terminal domain couples transcription and 3' end processing. Mol Cell *13*, 67-76.

Birse, C.E., Minvielle-Sebastia, L., Lee, B.A., Keller, W., and Proudfoot, N.J. (1998). Coupling termination of transcription to messenger RNA maturation in yeast. Science *280*, 298-301.

Dye, M.J. Gromak, N. and Proudfoot, N.J. (2006) Exonic tethering of RNA polymerase II transcripts. Molecular Cell, 21, 849-859 (2006)

El Hage, A., Koper, M., Kufel, J., and Tollervey, D. (2008). Efficient termination of transcription by RNA polymerase I requires the 5' exonuclease Rat1 in yeast. Genes & development *22*, 1069-1081.

Kawauchi, J., Mischo, H., Braglia, P., Rondon, A., and Proudfoot, N.J. (2008). Budding yeast RNA polymerases I and II employ parallel mechanisms of transcriptional termination. Genes & development *22*, 1082-1092.

Minvielle-Sebastia, L., Preker, P.J., and Keller, W. (1994). RNA14 and RNA15 proteins as components of a yeast pre-mRNA 3'-end processing factor. Science *266*, 1702-1705.

Morillon, A., Karabetsou, N., O'Sullivan, J., Kent, N., Proudfoot, N., and Mellor, J. (2003). Isw1 chromatin remodeling ATPase coordinates transcription elongation and termination by RNA polymerase II. Cell *115*, 425-435.

Torchet, C., Bousquet-Antonelli, C., Milligan, L., Thompson, E., Kufel, J., and Tollervey, D. (2002). Processing of 3'-extended read-through transcripts by the exosome can generate functional mRNAs. Molecular cell *9*, 1285-1296.

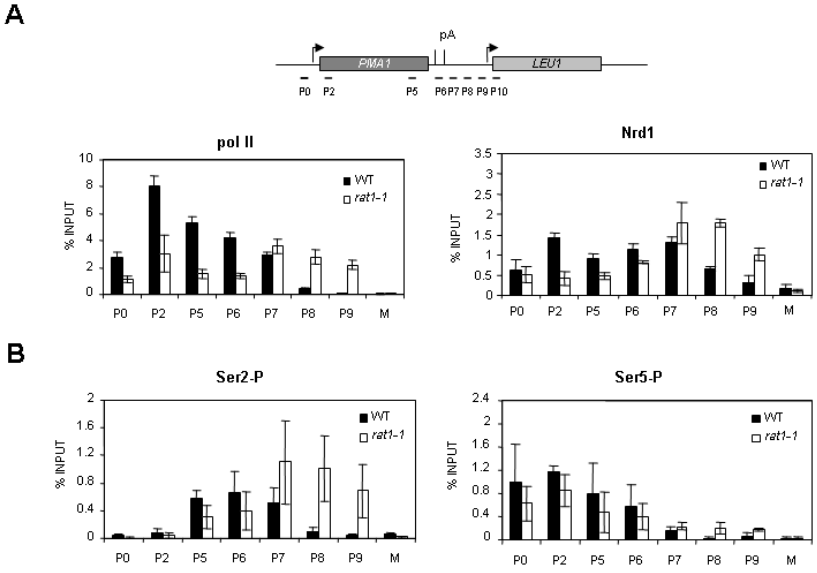

Suppl Fig. 1

Figure S1. *Nrd1 accumulates in PMA1 intergenic regions in rat1-1 mutants.*

(A) Diagram of *PMA1* and *LEU1*. Promoters denoted by arrows and pA signals by vertical lines. Horizontal bars denote the ChIP probes. ChIP analysis using either anti-Rpb1 (8WG16) or IgG fastflow agarose were performed in TAP-*NRD1 RAT1* (SC2290) or TAP-*NRD1 rat1-1* (SDY90B) strains. M corresponds to non-transcribed *MUC11*. Values shown represent average of three independent ChIP experiments and error bars show standard deviation. **(B)** ChIP assay using either anti-Ser2-P CTD (3E10) or anti-Ser5-P CTD (3E8) were performed in FD-4D (WT) or FD-4B (*rat1-1*).
